# Atlanta Society of Medicine

**Published:** 1898-04

**Authors:** 


### *SOCIETY REPORTS.*

### ATLANTA SOCIETY OF MEDICINE.

Atlanta, Ga., February 17, 1898.

Regular meeting of the Atlanta Society of Medicine.

The Society was called to order by the President at 8 P.M., and upon call of the roll the following members were present: Drs. Champion, Childs, Collins, Duncan, Felder, Hancock, Hoke, and Hurt.

The President then announced that the regular paper for the night was by Dr. Katherine Collins, on "Mentally Deficient Children." (To be published in this Journal.)

Dr. Collins read the paper, and the following discussion was had:

*The President:* You have heard this interesting paper by Dr. Collins. Dr. Duncan, we will be glad for you to open the discussion.

*Dr. Duncan:* It is one of the most interesting papers I have had the pleasure of hearing in this Society, bearing upon a most important subject, and Dr. Collins has shown that she has given the subject a great deal of thought, care and investigation. I do not know that I shall be able to say anything that will add to the interest of the subject. In the treatment of these cases the management of them, I think, should be commenced in early childhood, and especially so with the girls. I think they, at least, could be managed even in early life so as to be better prepared for living, and in later life, when girls are grown up and become married, during pregnancy they ought to receive the most careful attention. I think a great deal depends upon their impressions, upon what is brought to bear upon the mind of the mother during pregnancy. I think a great deal depends upon the acts and associations of the mothers. This, I think, is one cause why there are so many weak-minded children, the condition of the mothers during pregnancy, and I think this condition begins away back in girlhood in many cases—more especially in later life. I really believe that our educational system is a fraud in many respects. I believe that we have children in Atlanta who are suffering with literary dyspepsia, trying to crowd too many things into their minds in the public schools here. Take up any class in the schools and you will find that in one hundred pupils they are not all of the same ability, but they all have the same lessons. One-half of the pupils, perhaps, will learn the lesson well and may digest it well; consequently their minds grow stronger every day. The other half will not digest the lesson, and they have as much literary dyspepsia as a man would have dyspepsia if he overloaded his stomach with indigestible food. What we eat and digest gives us physical strength; what we eat and fail to digest injures us. Consequently I think there ought to be a different plan of education for the children. Take the kind of child that Dr. Collins mentions, a feeble-minded child who learns some things very well. I think if the child cannot get the lesson in a certain study that it ought not to be crowded. I don't believe that a child ought to be required to try to digest what it cannot digest. Consequently I do not think the educational system is as good here as if we had all these various classes recognized, and not try to crowd them in early life.

*Dr. Hurt:* I must compliment Dr. Collins upon the well-written paper. The statistics and the various points involved in the paper are of material importance at this present age of science in medicine, and, if I may be allowed to say so, science in society life or in social life. The question of weak minds, putting it in the most conservative and charitable light, and giving it that nomenclature, is affected by various influences. The influences that are brought to bear upon persons begin, in the judgment of some, from conception; and while in utero, and during early infancy and childhood, and on up during life, these influences are as varied as the surroundings and conditions under which life exists in such creatures. In utero, in my opinion, they are largely influenced by the state of the maternal mind during the period of gestation, and by the state of the physical motherhood as well. We all know that a sound and healthy female is prepared naturally for a healthy gestation and development of the fetus. We know equally as well that a feeble, nervous, sensitive, weakened physical condition is very much in the way of the development of the fetus physically. The same evil is very often transmitted to the nervous system and the mental functions or the mind, so we find children not infrequently coming into the world at the end of gestation who are under the ban of maternal influences which could not be avoided while in the state of health in which the mother was then existing. Again, it comes into life the victim of maternal impressions which doubtless might have been avoided and which ought to have been avoided. A physician is frequently handicapped in his efforts to take care of a female who has engaged him to attend her during parturition while he looks after her during gestation, because, while it is possible for him always to take cognizance of her surroundings, it is frequently impossible for him to get knowledge of all the conditons which attend her physically and mentally. Therefore, while he makes every effort, and well realizes that it is his duty to make every effort, to so control the mother in mind and body as to bring about a full development of the new-born at parturition, his efforts in this connection are often overcome by circumstances surrounding her which are beyond his control. Just this point occurred to me while listening to Dr. Collins on this subject, that the legislation looking to the care of these weak-minded and otherwise enfeebled persons, while the call for this has been so active, and very justly so, and it ought to be very much encouraged, there ought to be coupled with it legislation which will place as far as possible such environments around the mother, or the female expecting to become a mother, as will prevent such influences as are known or believed by many of us to be of irreparable damage to the fetus during the early months of gestation. To speak more plainly, the maternal impressions influencing the child in utero, in my mind, are unquestionably capable, not only of producing physical deformity, physical monstrosity, but of destroying all mental power, so that when the child is born, although it may have enough of nervous capacity to take charge of its physical part, it has no mental capacity of becoming really a citizen and occupying its place in the world. I believe that many of these imbeciles and weak-minded children are the direct results of maternal impressions. When we come to speak of maternal impressions, it is very necessary, if hard sometimes to do, to distinguish between maternal impressions which are calculated to be transmitted to the developing fetus and maternal impressions which go no further than simply to affect the woman herself. Now, if you will observe, or recall if you have had any experience in obstetrics, it is almost universally true that when a female is delivered one of the first questions is, "Doctor, is it all right?" This question goes to show that every woman bearing children has more or less dread or suspicion of some evil befalling the child in utero, and manifests her anxiety as soon as it is born by that question. The exaggerated impressions or exaggerated fear of such impressions is not always an index to what is going on to the damage of the child, but there are certain other influences, when closely inquired into, so convincing that no intelligent, thoughtful physician can for a moment doubt the certainty of such influences in utero. I think, if legislation could be had preventing the frequent exhibition upon our streets and over the country at large of the unfortunates—and among the unfortunates are just these classes that Dr. Collins has been talking about tonight, and along with these are those who are so physically deformed that their deformities become terrorizing to a woman in a delicate condition; and if such a woman passes along the street and comes in contact with such an object, she is first shocked, and the fright and shock are calculated to make impressions upon her mind so that the impressions are transmitted to the fetus or child in utero. Again, if she sees such an object frequently, or several times, she sometimes reports that at each successive time her fear and distress of mind has increased. I have known one woman so say, and voluntarily, too, that it oppressed her, took away her appetite, gave her a headache, made her sick and put her to bed. I have seen reports of other cases where abortion follows such a shock. Therefore, if the physical shock of such a thing could produce abortion, there is no reason why maternal impression should not obstruct or deform the developing fetus while growing in the uterus.

*Dr. Champion:* I don't know that I could add anything to what has already been said on this subject. I am satisfied that the Society appreciates Dr. Collins coming out to night and reading the paper. It is certainly a very interesting paper, and I am satisfied that this subject should be looked into more fully and carefully than it is. In all the schools in this country there are children who are mentally deficient, and not capable of keeping up with the classes. I think there ought to be schools especially for children of this kind. We have penitentiaries for criminals, we have lunatic asylums for the insane, and we have reformatories for criminals; but children of this class are placed in schools where they are not capable of competing with the other children. As Dr. Collins says, if they had a school or reformatory of some kind where they could be specially trained, they would amount to a great deal more in future life than they will otherwise. I am sorry that we have had such a bad night, so that we could not have more members out and have a full discussion.

*Dr. Collins:* I am glad that the doctor mentioned the education of the future mother. That is a very important subject, and I think the time will come when it will be a part of every girl's education, that it will be a part of the school system. In regard to the school system, I think the fundamental wrong is that there is too much work given to one teacher. When a teacher has fifty pupils to teach, she cannot study the individual capacity and individual traits of each child. Until the number is lessened, individual work cannot be done, and until it is done we cannot get the best results. Children are not so much taught too much, as they are taught in the wrong way. The doctor spoke of maternal impressions. I think that anything that will produce a shock will interfere to a certain extent with the general system, more especially with the nervous system. To what extent this will cause arrested development in the fœtus it is hard to say, but that it does cause a certain amount, I think is without doubt. I would not favor a reformatory for these children. We have had reformatories, and they mean punishment. These mentally deficients should be taken just as normal children are taken, except that we must begin with them a step lower. Teach them as a normal child is taught, on the same lines, only take a small amount of education at a time.

*Dr. Hurt:* Mr. President, I thought it might interest the Society to present this case. It is one that you do not see every day or anything like it, nor are we agreed as to the causes of such troubles or the remedies for such troubles. My object was more to illustrate and bring out a discussion upon the subject, looking to the benefit of the human race and the coming generations especially, and if I can so develop this idea as to bring forward into our legislative bodies at some future time the idea of the protection of females during gestation, I shall feel that my efforts have been well rewarded. I conscientiously feel like such things can be done and ought to be done, and that we are derelict in our duty until it is done. This is the case that I intend to present to-night. I was called, about the third of this month, I believe it was, to attend a lady twenty-five years old, who had been for over five years in the enjoyment of good health, and was well developed. Her husband was about thirty years old, the very picture of health, and a man of good size. This lady had miscarried at a period of some two or three years ago, I don't know exactly how long ago it was. That is the history that she gave me. Following the abortion she had an attack of abortive fever, which went into sepsis, and she had rather a severe spell, but that she had enjoyed very good health ever since she had got up from this sepsis. In making calculation as to her last menstrual period, she told me that the flow ceased on the 6th of June last. Having this data to calculate from as to the last menstrual period, my being called on the third of February shows that it must have been at least thirty days ahead of time. And from all the features of the case, I am prepared to say that she was delivered in the eighth month of gestation. The condition of the patient when I was called to see her was that of discomfort. She had a little headache, a little albumen in her urine, constipation and annoying, severe and irregular pains. I was summoned about eight o'clock in the morning with the announcement that she had been in labor all night. I was busy all day and didn't reach the house until about two o'clock in the afternoon. I made a digital examination, and directed that she be given an ounce of sulphate of magnesia, and told her that I would return at night unless I was summoned sooner. I returned about eight o'clock in the evening and found that very shortly after I had left the house the sac had ruptured. I found the os dilated to the size of a silver half dollar, and thought I detected what I was able to make out a vertex presentation, and I rested somewhat satisfied. But seeing that she was getting along slowly, and estimating that she would probably be in labor all night, I called in Dr. Corput, a young man who assists me sometimes, and told him to watch while I went and had some sleep. About two o'clock I was waked up. She was still having pains every few minutes. The pains were then every seven minutes. She complained of a nauseating headache, and her pulse was very high. I suggested to him to give her a hypodermic injection of five drops of Norwood's tincture of veratrum and this he did, and it had the result of removing the headache that she complained of. In two hours she was delivered, the pains recurring every seven minutes up to the time of delivery. They had not come any nearer together than seven minutes. At this hour, 2:30, I made a digital examination. There was some trouble with the presentation, and when I endeavored to relieve the matter of doubt, I found myself puzzled. I could not make out a satisfactory diagnosis of the presentation. Taking one view of it, I made it out a breech presentation, and taking another view of it, I thought that it was a brow presentation, but I made no satisfactory diagnosis. Sufficient to say, I found in the delivery, when the part presenting came down upon the perineum, it had in front of it a hard, spear-pointed substance tending to go directly into the perineum, immediately on the inside of which the point threatened laceration. To prevent this, I inserted my two fingers and held them under this point until it had passed through the vulva. After being passed through I announced to the mother that it was deformed, and while I was preparing to sever the cord, she told me that she had seen a man frequently on the streets during the early months of her pregnancy who had a large, red tumor on the back of his head, that extended down his neck and between his shoulders, with some long, red hairs on the sides of the tumor. If you will pay attention to her description of this man, and notice these pictures which I have brought here, you will see that that description fits this picture, except that this was bone tissue covered with a thin tissue of fibrous consistence enough to give it a red color, or scarlet color.

The strands of long, red hair are here identically as she described seeing them on the man on the streets. She said that each time she had seen him it had a terrible effect upon her, disturbing her sleep, loss of appetite, headache, and sick so that she would have to go to bed. I intended to bring the specimen with me and exhibit it to the Society, but the inclement weather prevented my getting it to the hall. It is in my office, and I shall be glad to show it to any of you if you care to call. This bony plate broadens until it is as wide as your two fingers and passes down over the neck and between the shoulders. The strands of hair are of a brown color, few and long, passing down on either side of this bony growth. Just as she described the hair on each side of the tumor. Then there is another story given by her husband. The front view, you see, is not at all normal, resembling a frog. You might say it resembles a frog or an ape or a brownie. He says that he and his wife were sitting down looking at some pictures of brownies, etc., on the back of a patent medicine sheet. And the discussion of these distorted brownies that they found there seemed to disturb her. While she herself made no mention of this, he called my attention to it at once, and I thought at the time, and now believe more fully, that this occurred and affected the fetus. So I am inclined to believe that this deformity of the fetus was caused from these compound maternal impressions, from seeing this man on the streets, and from the discussion of these pictures of brownies on the advertising sheet. The child was alive, at least she said she felt movement at the time the waters were discharging. When I reached her there was no sign of life. It was born dead. I am disposed to think, though I have never made a dissection of the subject, and I intend to make a thorough dissection and examination, but until I make the dissection and find out I am disposed to think that there was not enough development of the nervous system to bring about action of the heart and lungs.

*Dr. Childs:* Dr. Hurt, did she describe those facts before she saw it?

*Dr. Hurt:* She never has seen it. I told her husband that it was deformed and dead, and that I could not afford to let her see it, and asked him to let me take it away. She described the man she had seen on the streets of her own motion. As soon as she was delivered, I took it away into another room, and she never has seen it or any description of it.

*Dr. Collins:* I would like to know if you have had anything of her family history.

*Dr. Hurt:* I inquired into the family history on both sides as to whether there was any insanity, imbecility, neurotic tendencies, or anything departing from the normal, and I could not find anything in the history leading to any serious trouble in the family on either side.

---

### SCHLEICH'S ANESTHETIC MIXTURE.

Dr. Willy Meyer, in a letter to the *New York Medical Record* (December 4, 1897), recommends this anesthetic, after considerable experience with it at the German Hospital, New York. He regards it as safe. Schleich experimented with it on a physical and not on a chemical basis, adapting the boiling-point of the narcotic to the temperature of the body. He uses a mixture of three drugs, chloroform, ether, and petroleum ether (benzin—boiling-point 60° to 65° C. [140°—148° F.]).

Formula 1. Boiling point = 100.4° F.—Chloroform, 1½ oz.; petroleum ether, ½ oz.; sulphuric ether, 6 oz. Used in short operations.

Formula 2. Boiling point = 104° F.—Chloroform, 1½ oz.; petroleum ether, ½ oz.; sulphuric ether, 5 oz. For medium length operation.

Formula 3. Boiling point = 107.6° F.—Chloroform, 1 oz.; petroleum ether ½ oz.; sulphuric ether, 2⅜ oz. Used for major operations.

The measurement is made by volume, not by weight. It is a real solution, chemically.

He has used this mixture with the ordinary ether cone—paper and towel. The advantages of this anesthesia are: No cyanosis, salivation, or superabundant tracheal mucus; no bronchitis or broncho-pneumonia afterward. Effect on the heart less marked than that of chloroform.—*Am. Med. Surg. Bulletin.*

